# Vegfa/vegfr2 signaling is necessary for zebrafish islet vessel development, but is dispensable for beta-cell and alpha-cell formation

**DOI:** 10.1038/s41598-019-40136-1

**Published:** 2019-03-05

**Authors:** Chiara M. Toselli, Brayden M. Wilkinson, Joshua Paterson, Timothy J. Kieffer

**Affiliations:** 1 Department of Cellular and Physiological Sciences, Life Sciences Institute, University of British Columbia, Vancouver, BC V6T 1Z3 Canada; 2 Department of Surgery, Life Sciences Institute, University of British Columbia, Vancouver, BC V6T 1Z3 Canada

## Abstract

The mechanisms underlying zebrafish pancreatic islet vascularization have not been well characterized. We sought to determine the angiogenic factors responsible for islet vascularization and assess whether an absence of endothelial cells affects beta-cell and alpha-cell formation. We used a double transgenic zebrafish *Tg(fli1:EGFP; insa:tagRFP*) to label endothelial cells and beta-cells, respectively. Beta-cells developed adjacent to endothelial cells and by 72 hours post fertilization (hpf) the zebrafish pancreatic islet was highly vascularized. Zebrafish beta-cells express vascular endothelial growth factors *(vegf)*, *vegfaa* and *vegfab*. Double knockdown of *vegfaa* and *vegfab* or the primary Vegfa receptors (Vegfr2), *kdr* and *kdrl*, resulted in vessel deficient islets. While beta-cell and alpha-cell numbers remained unchanged in vessel deficient islets, *insulina* expression was downregulated relative to controls. Vegfaa/Vegfab-Vegfr2 signaling is necessary for proper islet vessel development, but not for the initial formation of beta-cells and alpha-cells.

## Introduction

The pancreas contains both endocrine and exocrine components. The exocrine pancreas constitutes the majority of the pancreas and produces digestive enzymes which are delivered to the duodenum. The endocrine pancreas consists of the islets of Langerhans that are scattered throughout the exocrine tissue. The primary function of pancreatic islets is to regulate blood glucose levels through the secretion of hormones. The islet consists of 5 endocrine cells types, the insulin secreting beta-cells, glucagon secreting alpha-cells, somatostatin secreting delta-cells, ghrelin secreting epsilon-cells, and the pancreatic polypeptide secreting PP-cells.

Pancreatic islets are highly vascularized. Studies in mice indicate that reciprocal interactions between endothelial cells and islets are important for proper islet development, maturation, and function^1,2^. During murine embryogenesis, endothelial cells are important in pancreas specification. The maintenance and induction of key pancreatic transcription factors PDX1 and PTF1A is dependent on signals from aortic endothelial cells, without which pancreas development is severely impaired^1–3^. In addition to initiating pancreas morphogenesis, endothelial cells also communicate with mature islet cells. These interactions between islet cells and endothelial cells are primarily mediated by vascular endothelial growth factor-A (VegfA) signaling^4^. Lack of islet VegfA in the early murine pancreas or in mature beta-cells results in a significant loss of intra-islet capillaries, impairments in insulin secretion, and glucose intolerance^4–8^.

While the role of endothelial cells on islet development has been well studied in murine models, it is less documented in zebrafish. Zebrafish is an ideal organism to study islet vessel development due to their transparency and rapid ex-utero development. Zebrafish pancreas development shares many similarities with mammals suggesting that studies within this system can have broadly relevant insights^9^. While it has been previously observed that some insulin-expressing cells still develop in *cloche* mutants which lack endothelial cells^10^, signals involved in zebrafish islet vascularization and its relationship with islet development is not completely understood.

In this study, we used a combination of genetic knockdown and pharmaceutical techniques to assess the role of *vegfaa* and *vegfab* in zebrafish islet vessel development and endocrine pancreas formation. We demonstrate that while Vegfaa/Vegfab-Vegfr2 signaling is necessary for proper islet vessel development, it is dispensable for the formation of both of the major islet endocrine cell types, beta-cells and alpha-cells.

## Results

### Endocrine pancreas is highly vascularized

**Figure 1:**
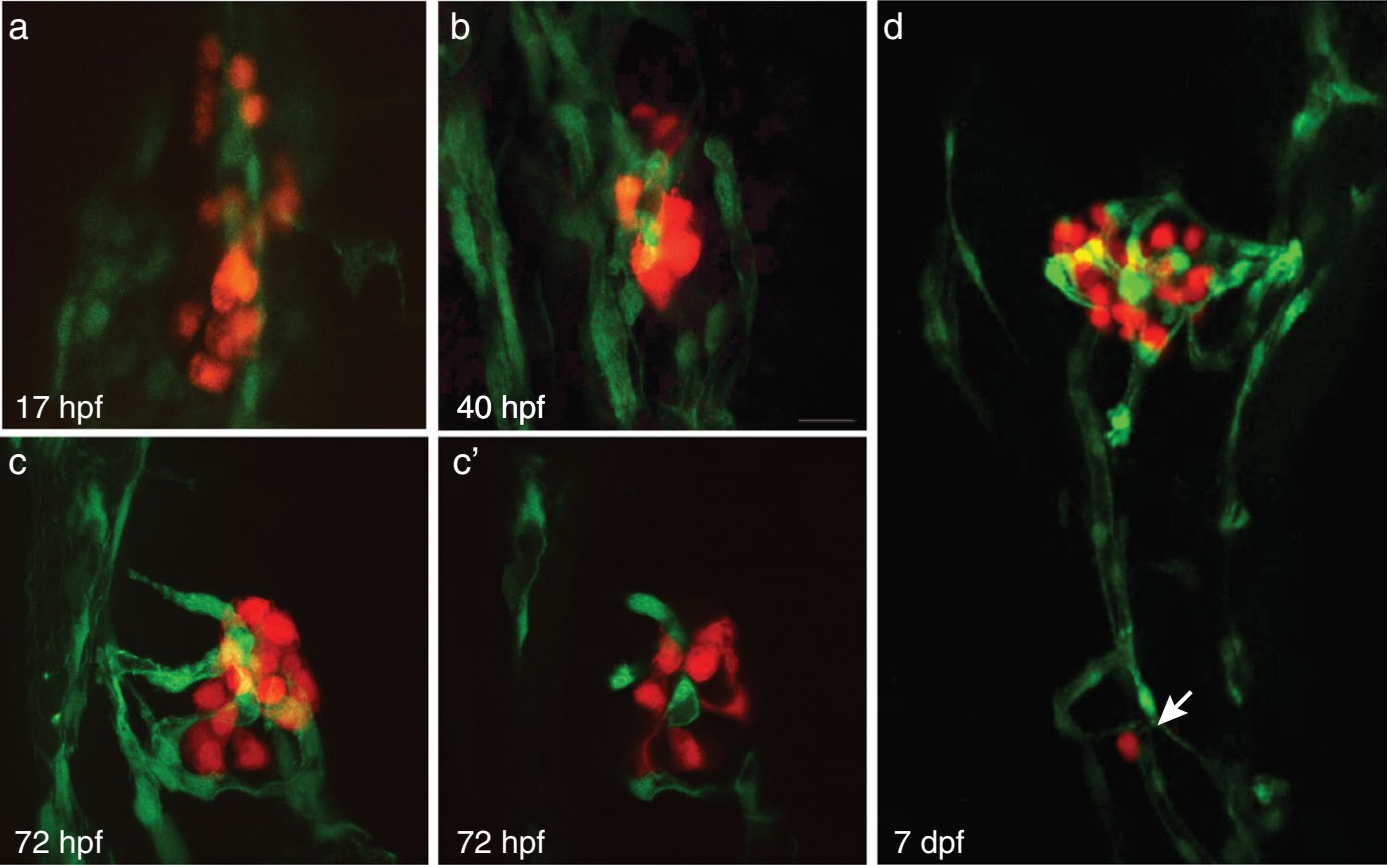
The endocrine pancreas develops adjacent to vessels and is highly vascularized. (**a–c**) Confocal projections of the pancreatic islet at 17 hpf, 40 hpf, and 72 hpf in *Tg(fli1:EGFP; insa:tagRFP);* endothelial cells (green) and beta-cells (red). (**c’**) Confocal section of projection in (**c**). (**d**) Confocal projection of 7 dpf *Tg(fli1:EGFP; insa:tagRFP)* pancreas. Arrow indicates secondary islet.

To characterize the formation of islet vessel development, we crossed *Tg(fli1:EGFP)* and *Tg(insa:tagRFP)* zebrafish to create a double transgenic line *Tg(fli1:EGFP; insa:tagRFP)* that labeled the endothelial/hematopoietic cells green and beta-cells red. Beta-cells developed adjacent to vessels at 17 hpf (Fig. 1a). As early as 40 hpf, endothelial cells were seen within the beta-cell core (Fig. 1b). At 72 hpf, the primary islet was highly vascularized in comparison to surrounding tissue (Fig. 1c). At 7 dpf, secondary islets were often observed adjacent to blood vessels (Fig. 1d).

### Vegf signaling is essential for islet vessel development, but not beta-cell and alpha-cell formation

**Figure 2:**
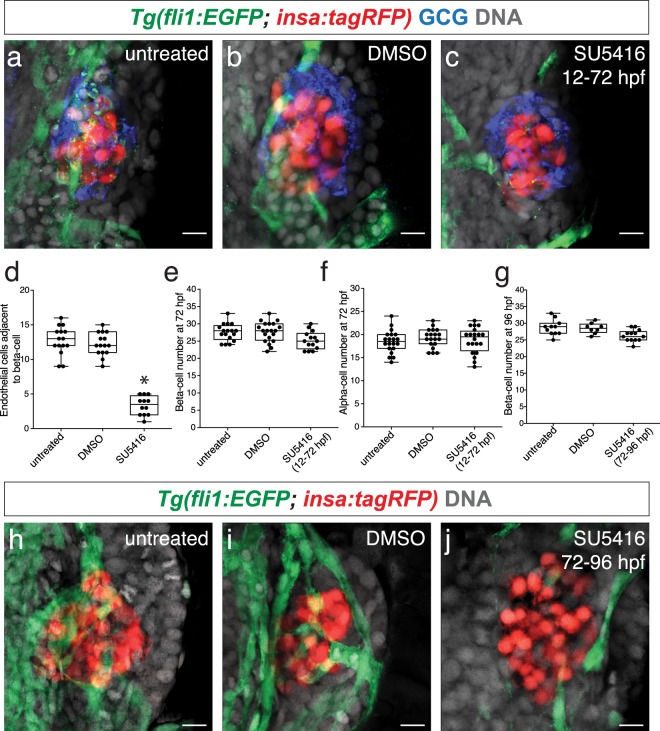
Inhibiting Vegf signaling does not affect beta-cell and alpha-cell formation. (**a–c**) Confocal projections of 72 hpf *Tg(fli1:EGFP; insa:tagRFP)* untreated, DMSO-treated, and SU5416-treated embryos from 12 to 72 hpf; endothelial cells (green), beta-cells (red), and DAPI nuclear stain (DNA; grey). Alpha-cells are labeled with a glucagon (GCG) antibody (blue). (**d**) The number of endothelial cells adjacent to beta-cells in untreated, DMSO-treated, and SU5416-treated embryos from 12 to 72 hpf. (**e**,**f**) The number of beta-cells and alpha-cells in *Tg(fli1:EGFP; insa:tagRFP)* untreated, DMSO-treated, and SU5416-treated embryos from 12 to 72 hpf. n = 14–20. (**g**) The number of beta-cells in *Tg(fli1:EGFP; insa:tagRFP)* untreated, DMSO-treated, and SU5416-treated embryos from 72 hpf to 92 hpf. n = 8–13. (**h–j**) Confocal projections of 96 hpf *Tg(fli1:EGFP; insa:tagRFP)* untreated, DMSO-treated, and SU5416-treated embryos from 72 to 96 hpf; endothelial cells (green), beta-cells (red), and DAPI (grey). (**d–g**) Box-and-whisker plots show median, and circles represent individual zebrafish. Scale bar = 10 μm.

To determine if Vegf signaling is required for islet vascularization, we administered a Vegf receptor competitive inhibitor SU5416. *Tg*(*fli1:EGFP; insa:tagRFP*) embryos were treated with SU5416 at 12 hpf until imaging at 72 hpf to assess the development of islet vessels. SU5416 treatment from 12 to 72 hpf reduced islet vessel density (Fig. 2a–d). This reduction in islet vessels may partially be caused by a failure of the sub-intestinal vein to form which partially gives rise to the pancreatic vessels^11^ (Supplementary Fig. 1a,b). No significant changes in beta-cell and alpha-cell numbers were observed in the SU5416-treated embryos (25.2 ± 2.6; 18.8 ± 2.8) in comparison to the DMSO-treated (27.5 ± 2.5; 19.2 ± 2.0) and untreated wildtype (27.7 ± 2.9; 18.5 ± 2.4) control embryos at 72 hpf (Fig. 2e,f). Islet architecture was not affected in vessel deficient embryos as the majority of alpha-cells were observed on the islet mantle with beta-cells localized to the islet core in both the control and SU5416-treated embryos.

To test if continued Vegf signaling is needed to sustain islet vessels, we treated embryos with SU5416 at 72 hpf until imaging at 96 hpf. We observed a reduction of islet vessels suggesting that continued Vegf signaling is necessary to sustain islet vasculature (Fig. 2h–j). No significant changes in beta-cell numbers were observed in these SU5416-treated embryos (26.3 ± 1.8) in comparison to DMSO-treated and untreated controls (28.5 ± 1.6; 28.7 ± 2.3) (Fig. 2g). We also administered SU5416 at 4.5 dpf until imaging at 6 dpf to determine if duct derived secondary islets form in vessel deficient fish. The proportion of fish that developed secondary islets did not change between SU5416-treated (21.4%) and DMSO-treated embryos (25.0%). (Supplemental Fig. 2a,b). We also examined secondary islet formation in SU5416 treated embryos given a γ-secretase Notch inhibitor (DAPT), which has been previously shown to stimulate the appearance of secondary islets in zebrafish^12,13^. From 3 dpf until 4.5 dpf, we administered DAPT or co-administered DAPT and SU5416 to *Tg(neuroD:GFP)* fish, a transgenic line that marks early pan-endocrine cells, thereby allowing us to capture early secondary islet formation. While there were no significant differences in secondary islet formation in the lower DAPT dose (20 μM) in comparison to untreated and DMSO-treated controls, we did observe a significant increase in the number of secondary islets with 100 μM DAPT (Supplementary Fig. 2d–j). The addition of SU5416 did not alter the effect of DAPT treatment on secondary islet formation (Supplemental Fig. 2d–j). These results suggest that Vegf signaling is dispensable for early secondary islet formation.

### Combined knockdown of *vegfaa* and *vegfab* causes a reduction of islet vessels

**Figure 3:**
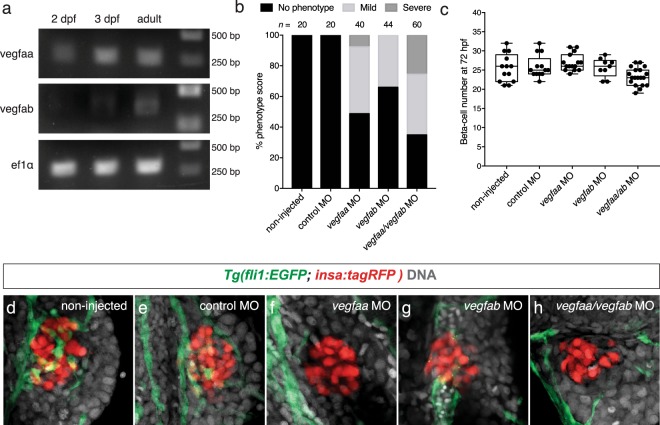
Vegfaa and Vegfab are necessary for islet vessel development. (**a**) RT-PCR of *vegfaa* and *vegfab* on sorted 2 dpf, 3 dpf, and adult beta-cells. Full length gel is presented in Supplementary Fig. 3. (**b**) Phenotypic score of islet vessels in non-injected, control morpholino and *vegfaa*, *vegfab*, or *vegfaa/vegfab* morpholino injected *Tg(fli1:EGFP; insa:tagRFP)* embryos. Phenotypes are scored such that no phenotype is comparable to wildtype (more than 7 endothelial cells adjacent to beta-cells), mild phenotype (4–7 endothelial cells adjacent to beta-cells), and severe phenotype (less than 4 endothelial cells adjacent to beta-cells). (**c**) The number of beta-cells in 72 hpf *Tg(fli1:EGFP; insa:tagRFP)* control and morpholino injected embryos. In the *vegfaa*, *vegfab*, and *vegfaa/ab* morpholino injected embryos, only the embryos that demonstrated a reduction or absence of islet vessels were counted. n = 9–21. Box-and-whisker plots show median, and circles represent individual zebrafish. (**d–h**) Confocal projections of (**d**) non-injected, (**e**) scrambled injected, (**f**) *vegfaa*, (**g**) *vegfab*, or (**h**) *vegfaa/ab* morpholino injected embryos at 72 hpf; endothelial cells (green), beta-cells (red), and DAPI (grey). Scale bar = 10 μm.

To identify potential mediators of signals responsible for islet vascularization, we isolated RFP-positive beta-cells from *Tg(insa:tagRFP)* larval fish and adult islets using fluorescence-activated cell sorting (FACS). We found that *vegfaa* was expressed in beta-cells isolated from fish at 2 dpf and 3 dpf. We also detected *vegfaa* and *vegfab* in adult isolated beta-cells (Fig. 3a and Supplemental Fig. 3a–c). We next injected previously validated translation blocking morpholinos against *vegfaa* and *vegfab* into *Tg*(*fli1:EGFP; insa:tagRFP)* embryos. In non-injected control zebrafish and those injected with scrambled control morpholinos, we observed greater than 7 endothelial cells adjacent to beta-cells, whereas those animals injected with morpholinos against *vegfaa* had either a mild reduction of islet vessels (between 4–7 endothelial cells adjacent to beta-cells) or a severe reduction of islet vessels (less than 4 endothelial cells adjacent to beta-cells) at 72 hpf (Fig. 3b,d–f). A small percentage of *vegfab* morpholino knockdown embryos exhibit a mild reduction in islet vessels (Fig. 3b,g). Combined knockdown of *vegfaa* and *vegfab* (*vegfaa*/*vegfab*) resulted in a more severe phenotype in comparison to the single knockdown embryos suggesting that both *vegfaa* and *vegfab* are important for islet vessel development (Fig. 3b,h). In the embryos that demonstrated a reduction or absence of islet vessels, no significant changes in beta-cell numbers were observed in these *vegfaa* (27.0 ± 2.2), *vegfab* (25.7 ± 2.5), and *vegfaa*/*vegfab* (23.3 ± 2.3) morpholino injected embryos in comparison to the scrambled injected or non-injected controls (25.9 ± 2.9; 25.5 ± 3.6) (Fig. 3c).

### Combined knockdown of *kdr* and *kdrl* causes a reduction of islet vessels

**Figure 4:**
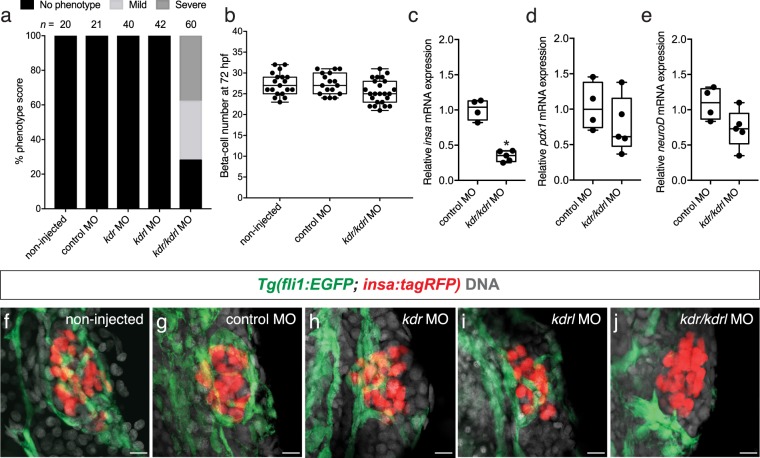
Knockdown of Vegfr2 receptors *kdr* and *kdrl* leads to disruptions in islet vessel development. (**a**) Phenotypic score of islet vessels in non-injected, control morpholino, and *kdr*, *kdrl*, or *kdr/kdrl* morpholino injected *Tg(fli1:EGFP; insa:tagRFP)* embryos. (**b**) The number of beta-cells in 72 hpf *Tg(fli1:EGFP; insa:tagRFP)* control and morpholino injected embryos. In the *kdr/kdrl* morpholino injected embryos, only the embryos that demonstrated a reduction or absence of islet vessels were counted. Box-and-whisker plots show median, and circles represent individual zebrafish. n = 17–24. (**c–e**) Relative expression of (**c**) *insa*, (**d**) *pdx1*, and (**e**) *neuroD* in isolated beta-cells of control injected and *kdr/kdrl* morpholino injected *Tg(fli1:EGFP; insa:tagRFP)* embryos at 72 hpf. All values were normalized to *ef1α*. *p < 0.0001 by Student’s t-test. (**f–j**) Confocal projections of (**f**) non-injected, (**g**) control injected, (**h**) *kdr*, (**i**) *kdrl*, or (**j**) *kdr/kdrl* morpholino injected embryos at 72 hpf; endothelial cells (green), beta-cells (red), and DAPI (grey). Scale bar = 10 μm.

We also assessed the role of *kdr* and *kdrl*, the primary receptors of VegfA in zebrafish. We found that single morpholino knockdown of either *kdr* or *kdrl* in *Tg*(*fli1:EGFP; insa:tagRFP)* zebrafish did not affect islet vessels (Fig. 4a,f–i). However, double knockdown of *kdr* and *kdrl* resulted in a reduction of islet vessels (Fig. 4a,j). In the *kdr/kdrl* injected embryos that demonstrated a reduction in islet vessels, no significant changes in beta-cell numbers were observed (25.5 ± 2.9) in comparison to the scrambled and non-injected controls (27.4 ± 2.6; 27.4 ± 2.7) (Fig. 4b).

To determine if beta-cells originating from the dorsal and ventral pancreatic bud form in vessel deficient fish, we performed a previously described label-retaining cell assay to mark dorsal pancreatic bud derived beta-cells^14^. In this assay, H2B-EBFP mRNA is injected into one cell stage embryos. The H2B-EBFP protein is diluted by cell division, but cells that are quiescent retain the label. The beta-cells originating from the dorsal pancreatic bud have been previously described to be quiescent by 24 hpf and retain the label (H2B-EBFP^+^). Ventral bud derived beta-cells arise later in development after multiple rounds of progenitor proliferation and consequently the ventral bud derived beta-cells do not retain the label (H2B-EBFP^−^). We found that both dorsal bud derived (H2B-EBFP^+^) and ventral bud derived beta-cells (H2B-EBFP^−^) are present in the *kdr/kdrl* injected embryos (Supplemental Fig. 4a–c). Together, these results suggest that Vegfaa/Vegfab-Vegfr2 signaling is necessary for islet vascularization but not required for beta-cell formation regardless of the origin of the beta-cells.

To determine if islet vessels influenced beta-cell maturation, we used FACS analysis to isolate RFP-positive *Tg*(*fli1:EGFP; insa:tagRFP*) beta-cells from scrambled injected controls and *kdr/kdrl* morpholino knockdown embryos at 72 hpf. Expression of *insulina (insa)* was significantly downregulated in vessel deficient embryos in comparison to scrambled injected controls (Fig. 4c), but levels of beta-cell maturation genes *pdx1* and *neuroD* were not significantly altered (Fig. 4d,e). We also observed a trending decrease in *insa* expression in SU5416-treated embryos compared to DMSO-treated controls (Supplemental Fig. 1c).

### A reduction of islet vessels is observed following beta-cell ablation

**Figure 5:**
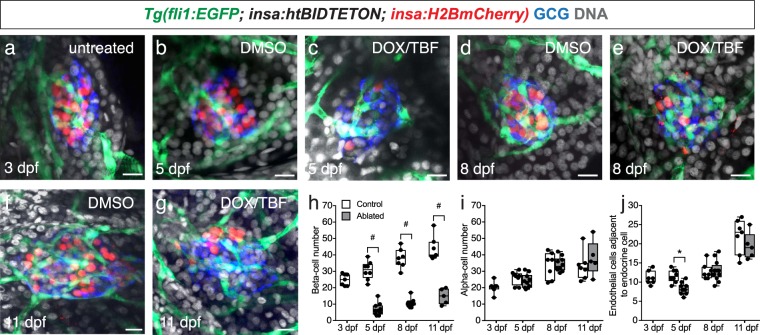
The number of islet endothelial cells decreases after beta-cell ablation. (**a–g**) Confocal projections of (**a**) 3 dpf, (**b**) 5 dpf control, (**c**) 5 dpf beta-cell ablated fish, (**d**) 8 dpf control, (**e**) 8 dpf beta-cell ablated fish, (**f**) 11 dpf control, (**g**) 11 dpf beta-cell ablated *Tg(*-*1*.*2ins:htBid*^*TE-ON*^; *-1*.*2ins:H2BmCherry; fli1:EGFP*) fish; endothelial cells (green), beta-cells (red), glucagon (blue) and DAPI (grey). Scale bar = 10 μm. (**h–j**) The number of (**h**) beta-cells, (**i**) alpha-cells, and (**j**) endothelial cells during beta-cell ablation and regeneration in *Tg(*-*1*.*2ins:htBid*^*TE-ON*^; *-1*.*2ins:H2BmCherry; fli1:EGFP*) fish treated with either DMSO (control) or DOX/TBF (beta-cell ablated) from 3–5 dpf. Box-and-whisker plots show median, and circles represent individual zebrafish. n = 5–12. Student’s t-test was conducted between the control and ablated groups at the same timepoint. #p < 0.0001, *p = 0.0004.

To determine if endothelial cells undergo changes during beta-cell ablation, we crossed the following double transgenic line, *Tg(*-*1*.*2ins:htBid*^*TE-ON*^; *-1*.*2ins:H2BmCherry)*^15^ to *Tg(fli1:EGFP)* to create the triple transgenic line *Tg(*-*1*.*2ins:htBid*^*TE-ON*^; *-1*.*2ins:H2BmCherry; fli1:EGFP)*. In this transgenic, the proapoptotic protein tBID is expressed under the control of the tetracycline- and ecdysone-inducible system which upon addition of DOX and TBF results in ablation of beta-cells^15^. We administered DOX and TBF (DOX/TBF) at 3 dpf until 5 dpf. We observed a significant decrease in the number of beta-cells following DOX/TBF administration at all timepoints in comparison to their DMSO-treated controls (Fig. 5a–h). There is no significant decrease in alpha-cell numbers in the DOX/TBF-treated fish in comparison to their timepoint matched DMSO-treated controls (Fig. 5i). We also observed a significant decrease in the number of endothelial cells at 5 dpf in the DOX/TBF-treated fish in comparison to DMSO-treated controls (Fig. 5a–c,j). However, there is no significant difference in the number of endothelial cells at 8 dpf or 11 dpf in DOX/TBF-treated fish in comparison to their timepoint matched DMSO-treated controls (Fig. 5d–g,j). These results suggest a decrease in the number of islet endothelial cells after beta-cell destruction, but revascularization of the islet during beta-cell regeneration.

## Discussion

In this study, we explored the angiogenic factors responsible for zebrafish islet vessel development and the effect of endothelial cells on pancreatic endocrine formation and maturation.

Beta-cells develop adjacent to blood vessels. Endothelial cells can be found within the islet as early as 40 hpf. The formation of beta-cells adjacent to endothelial cells is similarly observed in murine models where the pancreatic epithelium forms adjacent to embryonic endothelial cells^1^. Administration of the Vegf inhibitor SU5416 from 12 to 72 hpf caused a significant reduction of endothelial cells within the islet, although no significant changes in beta-cell and alpha-cell numbers were observed. Our results are consistent with previous studies which have observed the formation of insulin-expressing cells in vessel deficient *cloche* zebrafish mutants^10^. No significant changes in the number of secondary islets  were found in SU5416-treated fish from 4.5 until 6 dpf in comparison to control fish, suggesting that Vegf signaling is also dispensable for early secondary islet formation. Our results differ from murine pancreatic specification which is dependent on signals from the endothelial cells^1,2^ suggesting species differences in pancreas specification.

We found that treatment of zebrafish embryos with Vegf competitive inhibitor SU5416 from 72 hpf to 96 hpf after the islet vessels have been established leads to a decrease in islet vessels, suggesting that continuous Vegf signaling is needed to sustain islet vessels. This finding is consistent with observations in murine islets that Vegf signaling is the primary mediator of islet vessel formation and maintenance^4,5^.

To determine which Vegf angiogenic factors are responsible for zebrafish islet vessel development, we performed RT-PCR and found that both *vegfaa* and *vegfab* are expressed in the beta-cells as early as 2 dpf, suggesting the recruitment of endothelial cells early on during islet development. Combined knockdown of *vegfaa* and *vegfab* caused a more severe phenotype in the reduction of islet vessels in 72 hpf embryos in comparison to the single knockdown of *vegfaa* or *vegfab*. This may reflect partial redundancy between *vegfaa* and *vegfab*, as has been suggested for intestinal vessel development^16,17^. Despite the reduction of islet vessels in *vegfaa/vegfab* knockdown animals, there were no significant changes in beta-cell numbers between the vessel deficient and control islets. Similarly, double knockdown of the primary VegfA receptors, *kdrl* and *kdr*, resulted in a similar loss of islet vessels, but there were no significant changes in beta-cell numbers in *kdr/kdrl* knockdown animals in comparison to controls. Single knockdown of *kdr* or *kdrl* did not reduce islet vessels suggesting possible overlapping roles between the two receptors. These data are consistent with a previous report which found a more severe phenotype in intestinal vessel development when *kdrl*^um19^ mutants were also injected with a *kdr* morpholino in comparison to *kdrl*^um19^ mutants or *kdr* morpholino knockdown animals^17^. Together, these results suggest that VegfA/Vegfr2 signaling is necessary for islet vascularization but not required for beta-cell formation. In the vessel deficient embryos, we observed a decrease in *insa* mRNA expression in comparison to control animals, suggesting that islet hormone expression is influenced by Vegfa/Vegfr2 signaling. Similarly, in VegfA deficient mouse islets, a decrease in insulin expression has also been reported^18^. This reduction in insulin expression in the murine VegfA deficient islets is believed to be due to the absence of a vascular basement membrane which promotes insulin gene expression^18^.

In mice, VegfA inactivation during early development results in severe islet hypovascularization and significantly reduces beta-cell mass as a consequence of a decrease in beta-cell proliferation during postnatal stages^7^. While we did not observe any significant differences in beta-cell number between vessel deficient and control zebrafish at 72 hpf, this discrepancy may reflect the fact that beta-cell neogenesis and not proliferation is the main mechanism of beta-cell formation during zebrafish embryonic stages^14,19^. At 72 hpf, the majority of the islet is composed of quiescent dorsal bud derived beta-cells^14,20^. Thus, any putative changes in beta-cell proliferation would be undetected in our zebrafish model during early pancreas development. While there may be a decrease in beta-cell proliferation and mass during later stages of zebrafish pancreas development, an islet cell specific *vegfA* knockout zebrafish model may be needed to assess this as *vegfaa/ab* and *kdr/kdrl* knockdown models develop pericardial edema and die after 72 hpf, and SU5416 application in larval fish (after 72 hpf) for over 52 hours causes high rates of lethality (data not shown).

Oxygen accessibility may also lead to the species differences during pancreas development in vascular deficient models. In the murine pancreas, oxygen levels affect endocrine cell differentiation^21^. Embryos from pregnant rats exposed to a hypoxic environment (8% O_2_) prior to the secondary endocrine transition have significantly blunted endocrine differentiation in comparison to embryos from the control pregnant rats exposed to a normoxic environment (21% O_2_). *In vitro*, increasing oxygen levels (21%, 60%, and 80%) leads to the induction of endocrine hormone expression in cultured E13.5 rat pancreatic explants in a concentration-dependent manner^21^. As mammalian embryos require a functional cardiovascular system to bring oxygen to tissues, a lack of endothelial cells creates a hypoxic environment and consequently negatively affects endocrine pancreas differentiation. In contrast, given that zebrafish develop ex-utero, the embryos are in a normoxic milieu^22^. Hence, an avascular environment does not lead to hypoxia during early zebrafish development and may explain the formation of endocrine cells despite the lack of endothelial cells in our zebrafish models. Future studies controlling the ambient oxygen content could reveal its role during early endocrine cell formation in the zebrafish.

We also observed a decrease in islet endothelial cells after beta-cell destruction and revascularization of the islet during beta-cell regeneration. This is unlikely due to a bystander affect as alpha-cell number is not significantly reduced in beta-cell ablated fish compared to control fish, but rather this may indicate changes in vasculature organisation during beta-cell ablation. In the adult zebrafish pancreas, changes in islet vasculature after beta-cell destruction and during regeneration have been reported^23^. In other tissues, revascularization of the damaged tissue occurs quickly after insult^24,25^. In cardiomyocytes, this fast revascularization is essential in the regeneration process as it promotes cell proliferation^24^. Whether islet endothelial cells are needed for beta-cell regeneration is unknown. Future studies addressing beta-cell regeneration in an avascular islet environment may reveal novel mechanisms for beta-cell  renewal. In addition, identifying beta-cell transcriptome changes during beta-cell regeneration and during beta-cell development in an avascular environment could elucidate the role of endothelial cells in beta-cell formation and renewal.

In summary, the present study demonstrates that Vegfaa/Vegfab-Vegfr2 signaling is dispensable for beta-cell and alpha-cell neogenesis, although blocking Vegfaa/Vegfab-Vegfr2 signaling decreases *insulina* expression. Our study has identified Vegfaa and Vegfab as potential mediators for islet vessel development. These findings may have implications in zebrafish beta-cell regeneration studies, as we find changes in islet vasculature during beta-cell destruction and regeneration. Interfering with Vegfaa/Vegfab-Vegfr2 signaling during beta-cell regeneration may provide insight into mechanisms required for the endogenous expansion of beta-cell mass in zebrafish following beta-cell ablation.

## Methods

### Study Approval

All experiments were approved by the University of British Columbia Animal Care Committee and performed in accordance with the Canadian Council on Animal Care guidelines.

### Zebrafish Lines

Adult zebrafish were housed in a ZEBTEC zebrafish housing system (Tecniplast, Buguggiate, Varese, Italy) at 28 °C in a 14 hr light/10 hr dark cycle. Larvae and juvenile zebrafish (up until 1- month post fertilization) were fed with Gemma micro-150 (Skretting, Westbrook, ME, USA) once daily and artemia twice daily (Aquatic Eco-Systems, Apopka, FL, USA). Adults were fed Gemma micro-300 (Skretting) once daily and artermia (Aquatic Eco-Systems) twice daily. The following zebrafish lines were used in experiments: *Tg*(*fli1a:EGFP*)^26^ and *Tg(-1*.*2insa*:*tagRFP)*^27^, and *Tg(*-*1*.*2ins:htBid*^*TE-ON*^;*-1*.*2ins:H2BmCherry)*^15^. Fertilized eggs were collected from adult zebrafish and placed in E3 media (5 mM NaCl, 0.17 mM KCl, 0.33 mM CaCl_2_•2H_2_O, 0.33 mM MgSO_4_) at 28 °C in an incubator with a 14 hr light /10 hr dark cycle. After 8 hpf, embryos were treated with 0.003% 1-phenyl-2-thiourea (PTU) in E3 media to inhibit melanin pigment formation.

### Morpholino and mRNA Injections

Microinjection was performed using a MM-33 micromanipulator (Sutter Instrument, Novato, CA, USA) and a PicoSpritzer III pressure injector (Parker Hannifin Corp, Pine Brook, NJ, USA). Morpholinos were obtained from GeneTools LLC (Philomath, OR, USA). We injected previously described translation blocking morpholinos into one-cell stage embryos: 10 ng of MO1-vegfaa translation blocking (5′- GTATCAAATAAACAACCAAGTTCAT-3′)^28^, 3 ng of MO1-vegfab translation blocking (5′-GGAGCACGCGAACAGCAAAGTTCAT-3)^16^; 4.5 ng of MO2-kdr translation blocking (5′ - TATGCTCTATTAGATGCCTGTTTAA - 3′)^16^, and 4.5 ng of MO1-kdrl translation blocking (5′ - CCGAATGATACTCCGTATGTCAC - 3′)^29^. Our control was a standard scrambled morpholino (5′- CCTCTTACCTCAGTTACAATTTATA-3′) injected at 10 ng. All morpholino injections were repeated three independent times. For H2B-BFP mRNA injections, we cloned a T7 promoter upstream of a human histone 2B fused to EBFP2. The pEBFP2-H2B-BFP was a gift from Michael Davidson (Addgene plasmid, 55243). To generate mRNA, we used the mMESSAGE mMACHINE T7 Transcription as per the manufacturer instructions (Life Technologies, AM1344). We injected 100 pg H2B-BFP mRNA into the one cell stage of zebrafish embryos and kept the embryos at 28 °C in the dark until imaging.

### Chemical Treatments

SU5416 (Sigma-Aldrich, Oakville, ON, Canada S8442) was dissolved in DMSO to 40 mM and stored at −20 °C. Embryos were treated with 1 μM SU5416 or 0.1% DMSO in E3 media beginning at 12 hpf up until imaging at 72 hpf or at 72 hpf up until imaging at 96 hpf. The drug solution was refreshed every 24 hrs. To induce secondary islet formation, we administered N-[N-(3,5-Difluorophenacetyl-L-alanyl)]-S-phenylglycine *t*-Butyl Ester (DAPT) (EMD Millipore, 565770), γ-secretase inhibitor to block Notch signaling. DAPT was dissolved in DMSO to 10 mM and stored at −20 °C. The embryos were treated with 20 μM DAPT, 100 μM DAPT, or 1% DMSO in E3 media beginning at 3 dpf until imaging at 4.5 dpf. The embryos were kept 28 °C in the dark until imaging. To induce beta-cell ablation, larvae were treated with doxycycline hyclate (DOX) (Sigma-Aldrich D9891) at 100 mmol/l and tebufenozide (TBF) (EMD Millipore, 31652) at 50 mmol/l at 3 dpf until 5 dpf. Drugs were prepared as previously described^15^. The drug solution was refreshed after 24 hrs. The embryos were kept at 28 °C in the dark during DOX/TBF administration.

### FACS

We used 200 *Tg*(*fli1:EGFP; insa:tagRFP)* embryos to isolate RFP-positive beta-cells at 2 dpf and 3 dpf. The samples were dissociated in 0.25% trypsin at 37 °C for 30 min. The cells were filtered with a 40 μm nylon strainer. RFP-positive beta-cells were sorted using a BD Influx Cell Sorter (BD Biosciences, Mississauga, ON, Canada). For isolating adult beta-cells, pancreatic tissue was dissected from *Tg*(*insa:tagRFP*) fish and digested as previously described^30^. RFP-expressing beta-cells were sorted using BD Influx Cell Sorter (BD Biosciences).

### cDNA synthesis and RT-PCR

RNA was isolated from 500–2500 isolated RFP-expressing beta-cells using TRIzol (Invitrogen, Carlsbad, CA, USA). cDNA was prepared using the iScript cDNA synthesis kit (Invitrogen). For RT-PCR of vegfaa and vegfab, Accuprime Taq DNA Polymerase (Invitrogen) was used, and end products were run on a 1% agarose gel. For qPCR, cDNA was amplified with SsoFast EvaGreen Supermix (BioRad, Richmond, CA, USA) on a CFX96 Touch™ Real–Time PCR Detection System (BioRad). The relative expression of each sample was determined after normalization to *ef1α*. Primers for RT-PCR and RT-qPCR are listed: *vegfaa* forward 5′-AGAAAGAAAACCACTGTGAG-3′; *vegfaa* reverse 5′-AGGAATGTTCTTCCTTAGGT-3′; *vegfab* forward 5′- TGCTGAACACAGTGAA-TGCCAG-3′; *vegfab* reverse 5′- ACATCCATCTCCAACCACTTCAC-3′; *ef1α* forward 5′-TCAAGGACATCCGTCGTGGTA-3′; *ef1α* reverse 5′-ACAGCAAAGCGACCAAGAGG; *insa* forward 5′-TCTGCTTCGAGAACAGTGTG-3′; *insa* reverse 5′-GGAGAGCATTAAGGCCTGTG-3′; *pdx1* forward 5′-GGGCGCGAGATGTATTTGTTGA-3′; *pdx1* reverse 5′- CAAATCTCACACGCACGCATG-3′; *neuroD* forward 5′- TCATGCTTTCCTCGCTGTATGACT-3′; *neuroD* reverse 5′-CCACGAAGGGCATGAAACTATCA-3′. PCR cycling conditions were as follows: 3-min denaturation step at 95 °C, followed by 40 cycles of denaturation at 95 °C for 15 s, annealing at 55 °C for 15 s, and extension at 72 °C for 25 s.

### Immunofluorescence

Zebrafish embryos were anaesthetized with ethyl 3-aminobenzoate methanesulfonate (MS-222, 0.01% w/v) and then fixed with 4% paraformaldehyde overnight at 4 °C. Embryos were washed three times with 0.3% Triton X-100 in PBS. The embryos were blocked for 1 hr in PBS with 5% donkey serum (Jackson Immunoresearch Laboratories, West Grove, PA, USA) and 0.3% Triton X-100. Both primary and secondary antibodies were incubated overnight at 4 °C. Washes were done with 0.1% Tween/PBS for 30 min three times. Antibodies used were mouse anti-glucagon antibody (1:500, Sigma G2654) and goat anti-mouse Alexa Fluor 647 antibody (1:1000, Life Technologies A21236). Cell nuclei were visualized with 4′,6-diamidino-2-phenylindole (DAPI) DNA stain (1:1000, Thermo Fisher Scientific D1306) or TO-PRO™-3 Iodide stain (1:1000, Thermo Fisher Scientific T3605). Samples were mounted in 75% glycerol on concave glass slides (EISCO). All fluorescent images were obtained with a LSM800 Zeiss upright confocal and imaged with a 40X objective. Whole mount tissues were scanned by confocal microscopy. Cell numbers were counted manually. For endothelial cell counts, endothelial cells that were directly adjacent to islet cells (within 1 μm) were counted. In the DOX/TBF beta-cell ablation experiment, endothelial cells located within the islet or adjacent to alpha-cells (within 1 μm) were counted. Student’s t-test or one-way ANOVA and Tukey post-hoc test were used for statistical analysis for cell counts.

## Supplementary information

Supplementary Data

## Data Availability

All data generated or analysed during this study are included in this published article.
