# Supplementary material for: Vegfa/vegfr2 signaling is necessary for zebrafish islet vessel development, but is dispensable for beta-cell and alpha-cell formation
Source: Sci Rep. 2019 Mar 5;9:3594. doi: 10.1038/s41598-019-40136-1 (PMC6401103; doi:10.1038/s41598-019-40136-1)
Supplement: Supplementary file 1 — Supplementary Data [file 41598_2019_40136_MOESM1_ESM.pdf]

Supplementary Information

Vegfa/vegfr2 signaling is necessary for zebrafish islet vessel development, but is dispensable for beta- and alpha-cell formation

Chiara M. Toselli[1], Brayden M. Wilkinson[1], Joshua Paterson[1], Timothy J. Kieffer[1,2 *]

[1] Department of Cellular and Physiological Sciences, Life Sciences Institute, University of British Columbia, Vancouver, BC V6T 1Z3, Canada

[2] Department of Surgery, Life Sciences Institute, University of British Columbia, Vancouver, BC V6T 1Z3, Canada

* Corresponding Author: Timothy J. Kieffer (e-mail: tim.kieffer@ubc.ca)

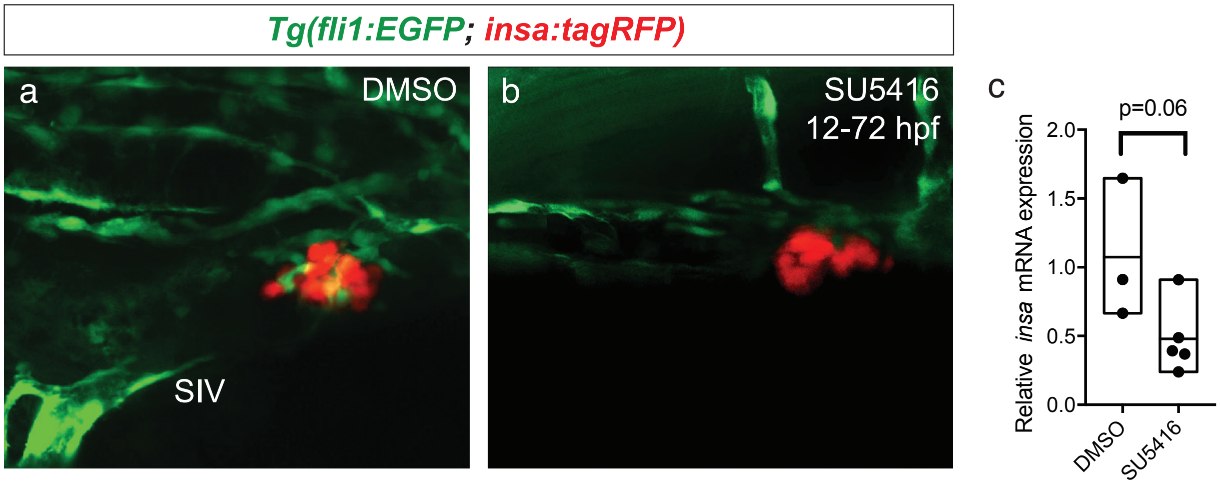

Supplemental Figure 1: Sub-intestinal vein fails to form in SU5416-treated embryos. (**a-b**) Confocal projections of 72 hpf *Tg(fli1:EGFP; insa:tagRFP)* embryos treated with DMSO or SU5416; endothelial cells (green) and beta-cells (red). SIV = sub-intestinal vein. (**c**) Relative expression of *insa* in isolated beta-cells of DMSO-treated and SU5416-treated *Tg(fli1:EGFP; insa:tagRFP)* embryos at 72 hpf. All values were normalized to *ef1α*.

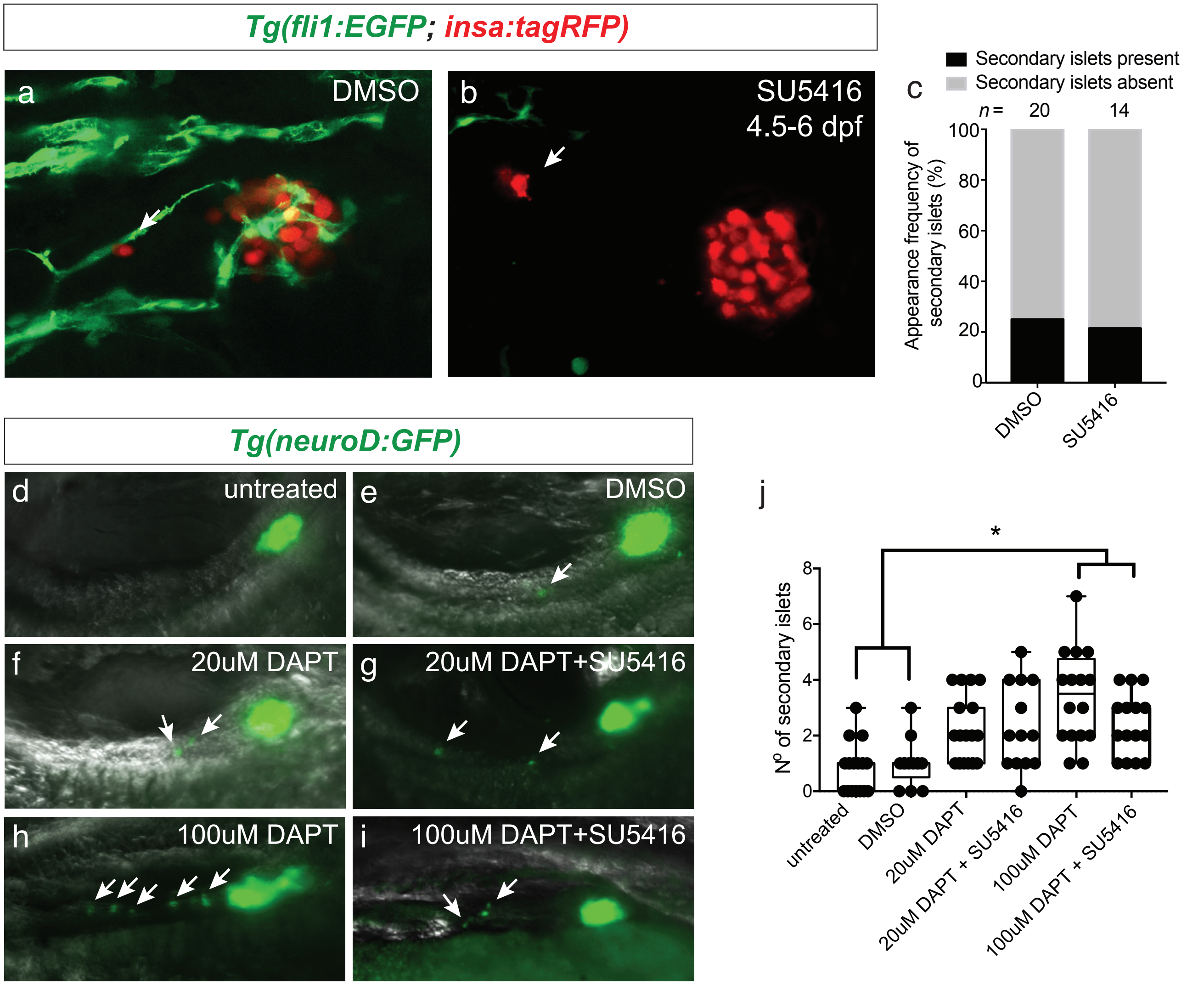

Supplemental Figure 2: Secondary islets form in SU5416-treated fish. (**a-b**) Confocal projections of 6 dpf *Tg(fli1:EGFP; insa:tagRFP)* zebrafish treated with DMSO or SU5416 from 4.5 dpf until 6 dpf. White arrowheads indicate secondary islets; endothelial cells (green) and beta-cells (red). (**c**) Frequency of secondary islets in 6 dpf *Tg(fli1:EGFP; insa:tagRFP)* zebrafish treated with DMSO or SU5416 from 4.5 dpf until 6 dpf. n=14-20. (**d-i**) Images of *Tg(neuroD:GFP)* untreated, DMSO-treated, DAPT-treated, or DAPT and SU5416-treated embryos from 3 until 4.5 dpf. White arrowheads indicate secondary islets. (**j**) The number of secondary islets in untreated, DMSO-treated, DAPT-treated, or DAPT and SU5416-treated embryos. Box-and-whisker plots show median, and circles represent individual zebrafish. n=13-19. *$p < 0.05$ by one-way ANOVA and Tukey post-hoc test.

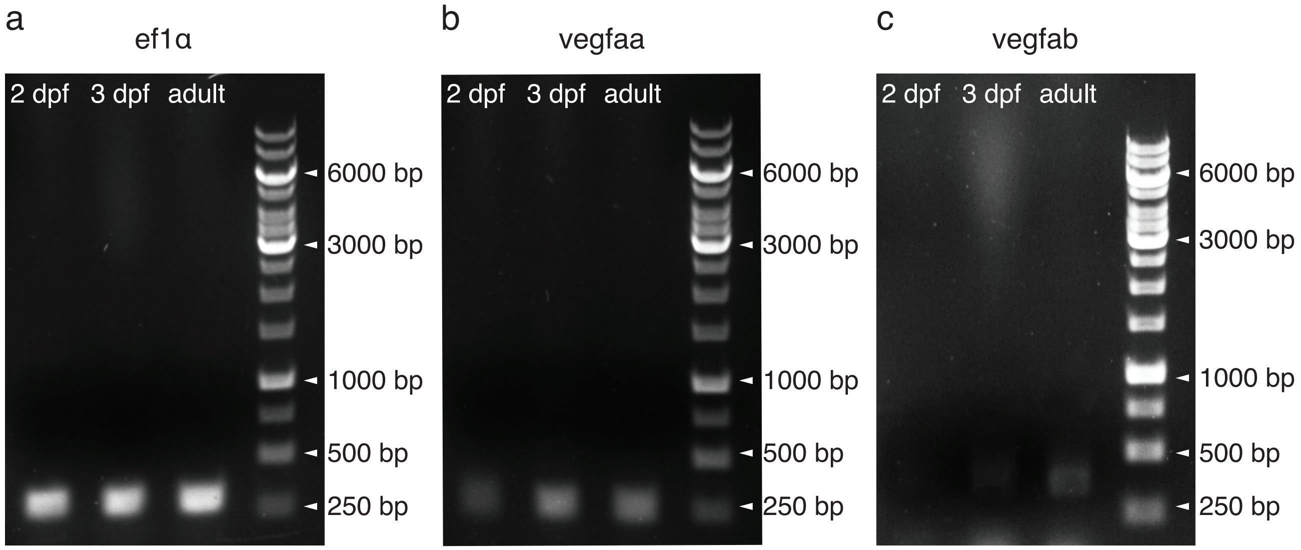

Supplemental Figure 3: Vegfaa and Vegfab expression in 2 dpf, 3 dpf, and adult beta-cells. (**a-c**) RT-PCR of (**a**) *ef1α*, (**b**) *vegfaa,* and (**c**) *vegfab* on sorted 2 dpf, 3 dpf, and adult beta-cells.

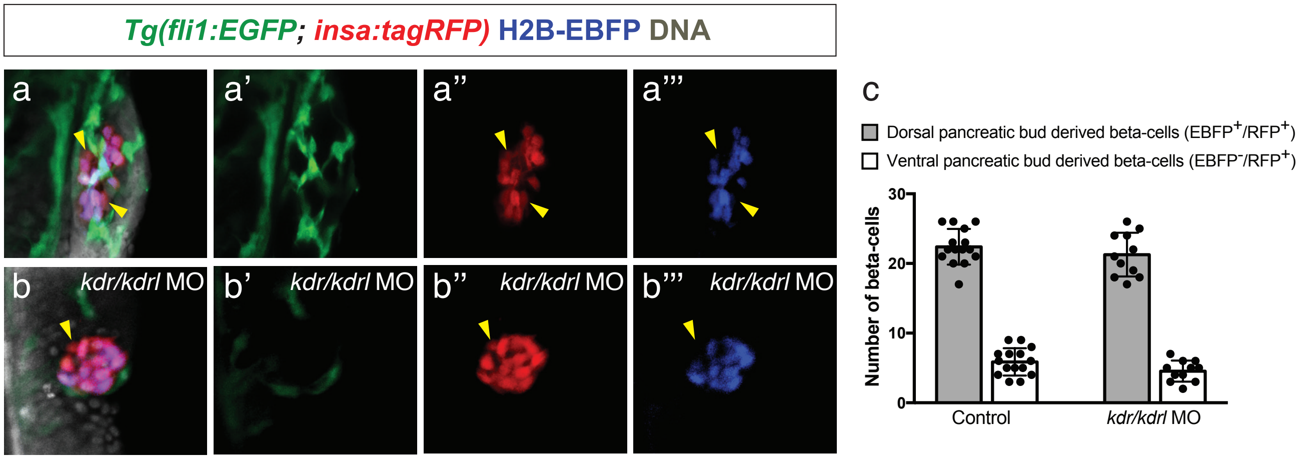

Supplemental Figure 4: Beta-cells derived from the dorsal and ventral pancreatic bud are present in *kdr/kdrl* injected zebrafish. (**a-b**''') Confocal projections of 72 hpf *Tg(fli1:EGFP; insa:tagRFP)* embryos (**a-a**''') injected with H2B-EBFP mRNA or (**b-b**''') co-injected with H2B-EBFP mRNA and *kdr/kdrl* morpholino; endothelial cells (green), beta-cells (red), dorsal pancreatic bud derived beta-cells (blue), and TO-PRO-3 nuclear stain (DNA; grey). Yellow arrowheads indicate beta-cells derived from the ventral pancreatic bud (H2B-EBFP$^-$/RFP$^+$). (**c**) The number of beta-cell derived from the dorsal pancreatic bud (H2B-EBFP2$^+$/RF-P$^+$) and ventral pancreatic bud (H2B-EBFP$^-$/RFP$^+$) at 72 hpf. Bar plots show mean, and circles represent individual zebrafish. n=11-15.
